# Nanoparticle-Encapsulated Curcumin Inhibits Diabetic Neuropathic Pain Involving the P2Y12 Receptor in the Dorsal Root Ganglia

**DOI:** 10.3389/fnins.2017.00755

**Published:** 2018-01-25

**Authors:** Tianyu Jia, Jingan Rao, Lifang Zou, Shanhong Zhao, Zhihua Yi, Bing Wu, Lin Li, Huilong Yuan, Liran Shi, Chunping Zhang, Yun Gao, Shuangmei Liu, Hong Xu, Hui Liu, Shangdong Liang, Guilin Li

**Affiliations:** ^1^Department of Physiology, Medical School, Nanchang University, Nanchang, China; ^2^Jiangxi Provincial Key Laboratory of Autonomic Nervous Function and Disease, Nanchang, China; ^3^Second Clinical Department, Medical School, Nanchang University, Nanchang, China; ^4^Department of Cell Biology, Medical School, Nanchang University, Nanchang, China

**Keywords:** nanoparticle-encapsulated curcumin, P2Y12 receptor, diabetic neuropathic pain, dorsal root ganglia, satellite glial cells

## Abstract

Diabetic peripheral neuropathy results in diabetic neuropathic pain (DNP). Satellite glial cells (SGCs) enwrap the neuronal soma in the dorsal root ganglia (DRG). The purinergic 2 (P2) Y12 receptor is expressed on SGCs in the DRG. SGC activation plays an important role in the pathogenesis of DNP. Curcumin has anti-inflammatory and antioxidant properties. Because curcumin has poor metabolic stability *in vivo* and low bioavailability, nanoparticle-encapsulated curcumin was used to improve its targeting and bioavailability. In the present study, our aim was to investigate the effects of nanoparticle-encapsulated curcumin on DNP mediated by the P2Y12 receptor on SGCs in the rat DRG. Diabetic peripheral neuropathy increased the expression levels of the P2Y12 receptor on SGCs in the DRG and enhanced mechanical and thermal hyperalgesia in rats with diabetes mellitus (DM). Up-regulation of the P2Y12 receptor in SGCs in the DRG increased the production of pro-inflammatory cytokines. Up-regulation of interleukin-1β (IL-1β) and connexin43 (Cx43) resulted in mechanical and thermal hyperalgesia in rats with DM. The nanoparticle-encapsulated curcumin decreased up-regulated IL-1β and Cx43 expression and reduced levels of phosphorylated-Akt (p-Akt) in the DRG of rats with DM. The up-regulation of P2Y12 on SGCs and the up-regulation of the IL-1β and Cx43 in the DRG indicated the activation of SGCs in the DRG. The nano-curcumin treatment inhibited the activation of SGCs accompanied by its anti-inflammatory effect to decrease the up-regulated CGRP expression in the DRG neurons. Therefore, the nanoparticle-encapsulated curcumin treatment decreased the up-regulation of the P2Y12 receptor on SGCs in the DRG and decreased mechanical and thermal hyperalgesia in rats with DM.

## Introduction

Type 2 diabetes mellitus (T2DM) is a metabolic disorder characterized by hyperglycemia (Whiting et al., 2011; Tesfaye and Selvarajah, 2012; Ma and Chan, 2013; Xu et al., 2013; Zychowska et al., 2013). Diabetic peripheral neuropathy is the most common complication of T2DM and results in sensory symptoms, including diabetic neuropathic pain (DNP) (Morales-Vidal et al., 2012; Singh et al., 2014). The etiology of DNP is characterized by hyperalgesia and allodynia (an increased sensitivity to painful or nonpainful stimuli, respectively) (Callaghan et al., 2012; Singh et al., 2014; Liu et al., 2016; Li et al., 2017; Peng et al., 2017). Low-grade inflammation is involved in the pathogenesis of diabetic peripheral neuropathy (Shoelson et al., 2006). Elevated levels of inflammation are associated with the development of DNP (Pop-Busui et al., 2016). The currently prescribed drugs are not satisfactory as treatments for DNP (Callaghan et al., 2012; Zychowska et al., 2013; Albers and Pop-Busui, 2014). Numerous studies have tried to develop improved pain-relieving treatments (Callaghan et al., 2012; Zychowska et al., 2013; Albers and Pop-Busui, 2014). Drugs that target low-grade inflammation may be useful for relieving DNP.

Adenosine triphosphate (ATP) activates purinergic 2 (P2) receptors involved in the signaling transmission of neuropathic pain (Liang et al., 2010; Lin et al., 2010; Illes et al., 2012; Katagiri et al., 2012; Li et al., 2012; Liu et al., 2012; Xu et al., 2012; Burnstock, 2013; Magni and Ceruti, 2013; Horvath et al., 2014; Idzko et al., 2014). P2 receptors consist of P2X (a ligand-gated ion channel receptor) and P2Y (a G protein-coupled receptor) (Katagiri et al., 2012; Burnstock, 2013; Magni and Ceruti, 2013; Horvath et al., 2014). In the dorsal root ganglia (DRG), satellite glial cells (SGCs) enwrap the neuronal soma (Hanani, 2005; Costa and Moreira Neto, 2015). The P2Y12 receptor is expressed on SGCs in the DRG (Katagiri et al., 2012; Kobayashi et al., 2013). The P2Y12 receptor is activated by ATP, and ADP plays an important role in the transmission of painful signals (Katagiri et al., 2012; Burnstock, 2013; Magni and Ceruti, 2013; Horvath et al., 2014). Both neurons and glial cells release ATP (Sperlagh et al., 1995; Vizi et al., 1997a,b; Fields and Burnstock, 2006). Peripheral inflammation increases SGC sensitivity to ATP in the primary sensory ganglia (Ceruti et al., 2008; Jasmin et al., 2010; Kushnir et al., 2011). Curcumin extracted from the natural medicine turmeric rhizome has anti-inflammatory and antioxidant properties and has been reported to ameliorate DNP (Li et al., 2013; Banafshe et al., 2014; Zhao et al., 2014). Nanoscale chemotherapy delivery system for treatment can increase the sensitivity of cells to drugs and reduce the side effects of therapy by targeting the action of drugs on the treated cells (Guo et al., 2016). Because curcumin has poor *in vivo* metabolic stability and low bioavailability (Anand et al., 2007; Guo et al., 2016), nanoparticle-encapsulated curcumin was used to improve its targeting and bioavailability. The aim of the present study was to investigate the effects of nanoparticle-encapsulated curcumin on DNP mediated by the P2Y12 receptor expressed on SGCs in the rat DRG.

## Materials and methods

### Animals and the type 2 diabetic rat model

Male Sprague-Dawley rats weighing 180–220 g were provided by the Center of Laboratory Animal Science of Nanchang University. Use of the animals was approved by the Animal Care and Use Committee of Medical College of Nanchang University. A quiet, good interior ventilated environment was provided for the rats. The room was maintained at 22°C with 50% humidity, a light illumination cycle of 12:12 h and free access to food. Cages and bedding were changed frequently. Rats were randomly divided into the following four groups: a control group, a type 2 diabetes mellitus (DM) group, DMs group treated with nanoparticle-encapsulated curcumin and DMs group treated with the nanoparticle-encapsulated carrier. The control group was fed a conventional diet (consisting of 5% fat, 53% carbohydrate, 23% protein, with a total calorie content of 25 kJ/kg), and the type 2 diabetic groups were fed a high-fat diet (consisting of 22% fat, 48% carbohydrate, and 20% protein with a total calorie content of 44.3 kJ/kg). The type 2 diabetic groups were intraperitoneally (i.p.) injected with a low dose of streptozotocin (STZ, Sigma, St. Louis, MO, USA) (30 mg/kg) in the fifth week (Peng et al., 2017). One week after the STZ injection, rats with fasting blood glucose levels > 7.8 mmol/L or non-fasting blood glucose levels > 11.1 mmol/L were considered diabetic rats. Control rats were administered the citrate buffer vehicle (pH 4.4) in a volume of 0.25 mL/kg (i.p.). Type 2 diabetic rats treated with nanoparticle-encapsulated curcumin were subjected to 2 injections of nanoparticle-encapsulated curcumin (16 mg/kg) in the sublingual vein in the 7th and the 8th weeks, and type 2 diabetic rats treated with the nanoparticle-encapsulated carrier received two injections with nanoparticle-encapsulated carrier (16 mg/kg) in the 7th and 8th weeks. There are 25% drug loading in the nanoparticles curcumin, so the actual dose of curcumin is 4 mg/kg. The nanoparticle-encapsulated curcumin and nanoparticle-encapsulated carrier were dissolved in normal saline. After the nanoparticle-encapsulated curcumin treatment, the blood glucose levels in type 2 diabetic rats were decreased compared with those in the untreated type 2 diabetic rats (see Table 1). There was no significant change in body weight between two groups.

**Table 1:** **(A)** Effect of the nano curcumin on blood glucose levels in type 2 diabetic rats. **(B)** Effects of nano curcumin on body weight (g) in type 2 diabetic rats.

**(A)**				
**Group**	**Blood glucose (mmol/L)**			
	**0 w**	**5 w**	**7 w**	**10 w**
Ctrl	5.33 ± 0.20	5.58 ± 0.33	5.67 ± 0.27	5.53 ± 0.46
DM	5.87 ± 0.34	13.85 ± 1.23^***^	15.03 ± 1.12^***^	14.86 ± 1.15^***^
DM+nano curcumin	6.09 ± 0.19	13.76 ± 1.11^***^	15.34 ± 0.84^***^	12.01 ± 0.93^***^^#^
DM+nano carrier	6.00 ± 0.32	13.03 ± 1.03^***^	14.63 ± 1.01^***^	14.58 ± 1.12^***^
**(B)**				
**Group**	**Body weight (g)**			
	**0 w**	**5 w**	**7 w**	**10 w**
Ctrl	197.88 ± 8.76	310.02 ± 17.09	372.39 ± 23.93	452.78 ± 25.38
DM	198.74 ± 9.38	342.32 ± 18.28^*^	347.92 ± 22.32^*^	304.34 ± 29.02^**^
DM+nano curcumin	197.51 ± 10.02	334.39 ± 20.02^*^	340.37 ± 21.78^*^	310.25 ± 27.33^**^
DM+nano carrier	196.89 ± 9.79	338.94 ± 19.39^*^	337.27 ± 25.03^*^	301.31 ± 28.20^**^

**(A)** Data are expressed as means ± SEM. The significant difference was denoted as

*** when p < 0.001 compared with the control group, and

# when p < 0.05 compared with the DM group. **(B)** Data are expressed as means ± SEM. The significant difference was denoted as

* when p < 0.05 and

** *when p < 0.01 compared with the control group*.

### Synthesis of poly-PEGMA-DMAEMA-MAO nanoparticle-encapsulated macromolecules

Biological nanoparticle-encapsulated carriers not only improve the solubility and bioavailability of a drug but also control drug release and attenuate toxic side effects (Bala et al., 2005; Bisht et al., 2007). Reversible addition fragmentation chain transfer (RAFT) radical polymerization has developed into an extremely versatile controlled/living free radical polymerization technique with respect to the reaction conditions and the wide range of applicable monomers (Liu et al., 2012). A typical protocol for the synthesis of an amphiphilic polymer macromolecule (PEGMA-DMAEMA-MAO) was conducted using RAFT solution polymerization (Byard et al., 2017). PEGMA served as the hydrophilic segment, MAO served as hydrophobic blocks, and DMAEMA served as a segment to bind DNA. Briefly, CTA (0.5 mmol), AIBN (0.05 mmol), DMAEMA (5 mmol), PEGMA (10 mmol), and MAO (10 mmol) were weighed into a 50-mL round-bottom flask. Methylbenzene (20 mL) was added to produce a homogeneous solution, which was purged with nitrogen for 30 min. The sealed flask was immersed in a 55°C oil bath for 24 h under a nitrogen atmosphere. Then, the polymerization reaction was subsequently dried under vacuum rotary steam. The block copolymer was purified by dialysis against distilled water/ethanol for 48 h (MWCO 3500) and recovered by freeze-drying. Purified PEGMA-DMAEMA-MAO was obtained as a yellow solid.

### The synthesis of curcumin loaded poly-PEGMA-DMAEMA-MAO microspheres

Curcumin-polybutylcyanoacrylate nanoparticle-encapsulated particles were prepared using anionic emulsion polymerization to combine the advantages of biological nanoparticle-encapsulated carriers and the beneficial effects of curcumin (Gaspar et al., 1992; Bisht et al., 2007; Shaikh et al., 2009). A typical oil-in-water (O/W) solvent evaporation method was used to prepare the microspheres (Al-Maaieh and Flanagan, 2001). Curcumin (50 mg) was dissolved in 16 mL of absolute ethyl alcohol. Poly-PEGMA-DMAEMA-MAO (60 mg) was dissolved in 6 mL of acetone. Then, the two solutions were mixed to obtain two-phase organic solvents. The organic solvents were dropped in distilled water (100 mL, pH 5.0) at a rate of 2 mL/min in an ice bath, and the organic phase was emulsified with a homogenizer into the aqueous phase. The dispersion was then evaporated under ambient temperature and pressure to harden the microspheres. Microspheres were then separated by filtration, vacuum-dried, weighed and stored in a vacuum desiccator.

### Measurement of the mechanical withdrawal threshold

The mechanical withdrawal threshold (MWT) was measured at 8:00–12:00 using a BME-404 electronic mechanical stimulator (Institute of Biomedical Engineering, Chinese Academy of Medical Sciences, Tianjin, China) (Lin et al., 2010; Li et al., 2017). The main technical parameters of this equipment were an end face diameter of the test needle of 0.6 mm, a pressure measurement range of 0.1–50 g, and a pressure measurement resolution of 0.05 g. An organic glass box (22 × 22 × 12 cm) was placed on the sieve of the metal frame. The rat was placed into the box for 30 min of adaptation. The left hind paws were touched with the test needle until an escape behavior was observed. The pressure on the value was automatically recorded. Measurements were performed 5 times for each rat (interval ≥ 5 min), and the mean value was calculated as the MWT for this measurement (Lin et al., 2010; Li et al., 2017).

### Measurement of the thermal withdrawal latency

The thermal withdrawal latency (TWL) was determined by exposing the plantar surface of the hind paw to radiant heat using the Thermal Paw Stimulation System (BME-410C, Tianjin) (Lin et al., 2010; Li et al., 2017). Rats were placed in a transparent, bottomless acrylic box on a glass plate with a light located underneath it. After a 30-min habituation period, the plantar surface of the paw was exposed to a beam of radiant heat applied through the glass floor. The light beam was switched off when the animal lifted its paw. The cutoff time for heat stimulation was 30 s. The hind paws were tested by a blinded observer in triplicate at 5-min intervals.

### Quantitative real-time PCR

The rats in the 4 groups were anesthetized with 10% chloral hydrate (3 mL/kg, i.p.). DRGs were isolated immediately and flushed with ice-cold PBS. Total RNA samples were prepared using TRIzol Total RNA Reagent (Beijing Tiangen Biotech Co.). The cDNA synthesis reaction was performed with 2 μg of total RNA and the RevertAid™ H Minus First Strand cDNA Synthesis Kit (Fermentas, Burlington, Ontario, Canada). Primers were designed with Primer Express 3.0 software (Applied Biosystems), and the sequences were as follows: Quantitative real-time PCR (qPCR) was performed using the SYBR® Green MasterMix in an ABI PRISM® 7500 Sequence Detection System (Applied Biosystems, Inc., Foster City, CA). Gene expression was quantified using the ΔΔCT method with CT as the threshold cycle. The relative levels of target genes, which were normalized to the sample with the lowest CT, were reported as 2^−ΔΔCT^.

### Double-labeled immunofluorescence staining

Double-label immunofluorescence staining was performed as previously described. Briefly, DRGs were removed from the rats and fixed with 4% paraformaldehyde (PFA) for 2 h at room temperature. DRGs were dehydrated in 30% sucrose and 4% PFA overnight. Then, 10-μm-thick sections were cut using a cryostat and mounted on slides. Sections were washed with PBS and incubated in a blocking solution containing 3% bovine serum albumin (BSA) and 0.3% Triton X-100 in PBS for 30 min at room temperature. Primary antibodies against glutamine synthetase (GS) (mouse anti-GS, Abcam) were diluted 1:100, mouse monoclonal anti-calcitonin generelated peptide (CGRP) (1:150 dilution, Abcam, USA), and the P2Y12 antibody (rabbit anti-P2Y12, Abcam) was diluted 1:200. Sections for P2Y12 and GS were washed again with PBS and incubated with TRITC-conjugated goat anti-rabbit (1: 2000, Jackson ImmunoResearch, Inc., West Grove, PA, USA) and FITC-conjugated goat anti-mouse secondary antibodies (1: 2000, Jackson ImmunoResearch, Inc., West Grove, PA, USA), and sections for P2Y12 and CGRP were washed again with PBS and incubated with TRITC-conjugated goat anti-mouse (Abcam, 1:2000) and FITC-conjugated goat anti-rabbit (Abcam, 1:2000) in PBS for 1 h at 37°C. Finally, sections were washed with PBS, and the results were assessed under a fluorescence microscope (Olympus, Tokyo, Japan). Data were collected from seven animals in each group. Four fields were randomly selected, and data from each animal were averaged.

### Western blot analysis

DRGs were removed and stored at −80°C. The ganglia were homogenized in RIPA lysis buffer [50 mM Tris-Cl, pH 8.0, 150 mM NaCl, 0.1% sodium dodecyl sulfate (SDS), 1% Nonidet P-40, 0.02% sodium deoxycholate, 100 μg/mL phenylmethylsulfonyl fluoride, and 1 μg/mL aprotinin] containing a protease inhibitor cocktail. Ganglia were incubated on ice for 30 min and subsequently centrifuged (12,000 g for 10 min). The supernatants were collected and protein concentrations were determined using a BCA Protein Assay Kit. These supernatants were diluted with 6 × loading buffer and heated to 95°C for 5 min; aliquots containing 20 μg of protein from each group were then separated using a Bio-Rad 10% SDS–polyacrylamide gel electrophoresis system and transferred to polyvinylidene fluoride (PVDF) membranes. The membrane was blocked with 5% non-fat dry milk in 1x TBST for 2 h at room temperature, followed by an incubation with rabbit anti-P2Y12 (1:1,000 dilution, Abcam, USA), rabbit anti-interleukin-1β (IL-1β) (1:800 dilution, Abcam, USA), rabbit anti-connexin43 (Cx43) (1:800 dilution, Abcam, USA) rabbit anti-phosphorylated-Akt (p-Akt) (1:500 dilution, Cell Signaling Technology, USA) rabbit anti-Akt (1:500 dilution, Cell Signaling Technology, USA), and mouse monoclonal anti-β-actin antibodies (1:800 dilution, Beijing Zhongshan Biotech Co., China) overnight at 4°C. The membrane was washed three times with TBST and incubated with a horseradish peroxidase-conjugated secondary antibody (goat anti-rabbit IgG and goat anti-mouse IgG, 1:2,000 dilution, Beijing Zhongshan Biotech Co., China) in blocking buffer for 1 h at room temperature. After another wash cycle, the labeled proteins were visualized by enhanced chemiluminescence (ECL) using a Bio-Rad system. Band intensity was quantified using Image-Pro Plus software. The integrated optical density (IOD) of each band was quantified using Image-Pro Plus software. The IOD of the target proteins was normalized to the intensity of the respective β-actin internal control.

### Molecular docking

Molecular docking computations were performed using AutoDock 4.2 (Morris et al., 2009; Trott and Olson, 2010). Molecular docking is a computer simulation tool that attempts to predict the binding mode of a ligand in the active site of a protein. Molecular docking studies mimic the natural interaction of a ligand with the protein. The docking technique was designed to position the ligand in different orientations and conformations within the binding site to calculate optimal binding geometries and energies. Therefore, after the docking procedure, the proper conformation of the ligand in the active site of the protein is obtained and used to calculate molecular descriptors. For each ligand, a number of configurations called poses are generated and scored (Trott and Olson, 2010). The score is calculated as either a free energy of binding, which considers solvation and entropy, the enthalpic term of the free energy of binding, or a qualitative shaped-based numerical measure. The final top-scoring poses, along with their scores and conformation energies, were input into a database for further analysis.

Protein Data Bank entry NP_073637 was used as the target protein (Biasini et al., 2014). Curcumin, PubChem CID 969516, was used as the ligand. Both structures were prepared using AutoDockTools (ADT) (Morris et al., 2009) and Python scripts named prepare_ligand4.py and prepare_receptor4.py, which are associated with the AutoDock4.2 program. The binding pocket position in the target protein was specified with the ADT molecular viewer. The parameters were maintained at their default values. Finally, the output files were viewed using MGLtools (Morris et al., 2009) and PyMol (http://www.pymol.org/).

### Statistical analysis

The data were analyzed using SPSS 20 software. The numerical values were reported as the mean ± SEM. Statistical significance was determined by one-way analysis of variance followed by the Fisher's *post-hoc* test for multiple comparisons. A *p* < 0.05 was considered statistically significant.

## Results

### Effects of nanoparticle-encapsulated curcumin on mechanical or thermal hyperalgesia in DM rats

**Figure 1:**
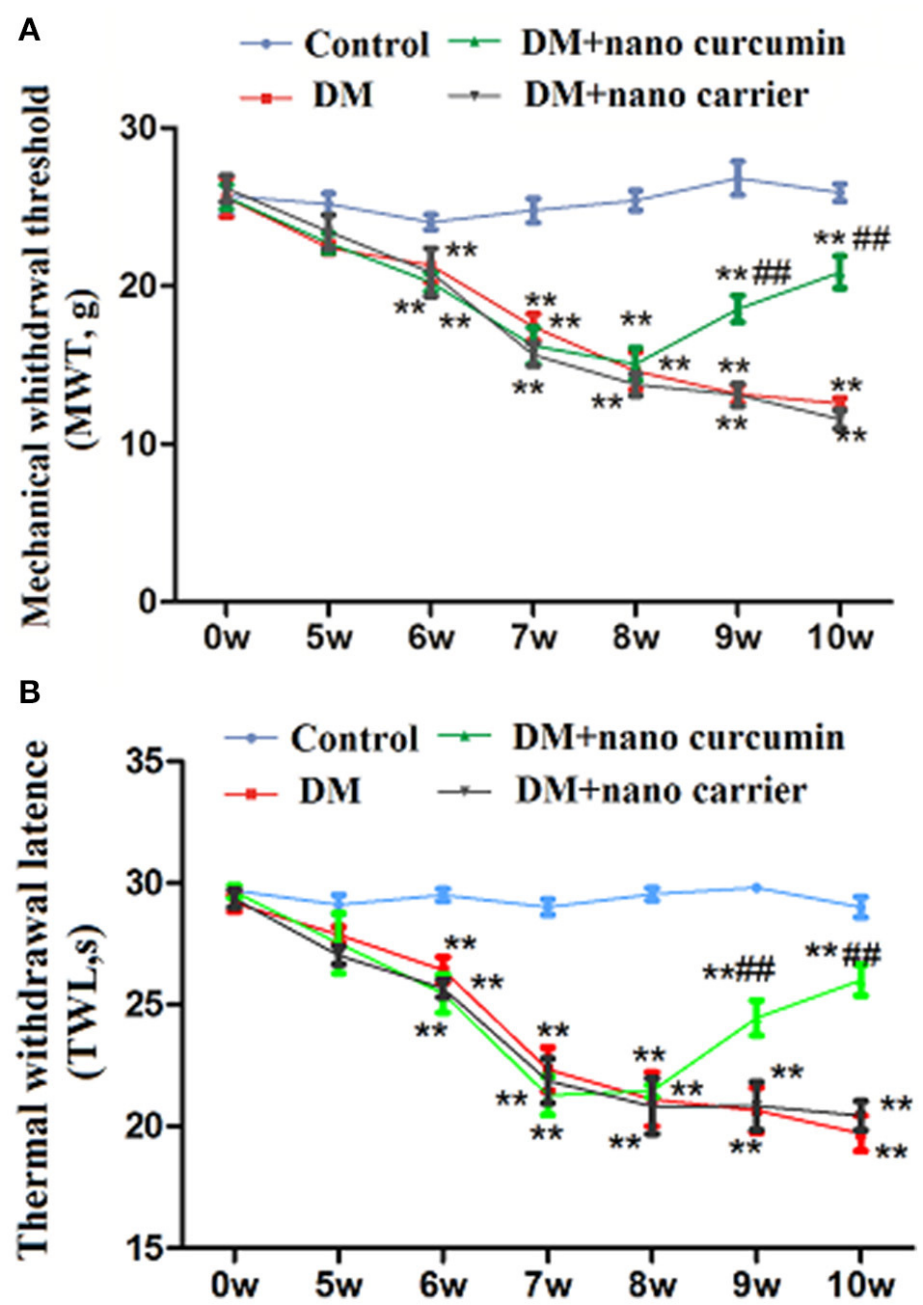
Effects of nanoparticle-encapsulated curcumin on mechanical or thermal hyperalgesia in DM rats. **(A)** The MWT in the DM group was lower than that in the control group. The MWT was higher in DM rats treated with nanoparticle-encapsulated curcumin than that in the untreated DM group. No difference was observed between the DM+nanoparticle-encapsulated carrier group and the DM group (*p* > 0.05). Each group comprises seven rats. Data are displayed as means ± SEMs. ^**^p < 0.01 compared to the control group; ##*p* < 0.01 compared to the DM group. **(B)** The TWL in the DM group was lower than that in the control group. The TWL was higher in DM rats treated with nanoparticle-encapsulated curcumin than that in the untreated DM group. No difference was observed between the DM+nanoparticle-encapsulated carrier group and the DM group (*p* > 0.05). Each group comprises seven rats. Data are displayed as means ± SEMs. ^**^p < 0.01 compared to the control group; ## *p* < 0.01 compared to the DM group.

The MWT was tested with a mechanical stimulator. A significant difference was observed from the 6th week to the 10th week (*p* < 0.01) between the control group and the DM group. At the 10th week, the TWL in the DM+nanoparticle-encapsulated curcumin group was higher than that in the DM group (*p* < 0.01). No difference was observed in the MWT between the DM and the DM+nanoparticle-encapsulated carrier groups (*p* > 0.05; Figure 1A). Based on these results, nanoparticle-encapsulated curcumin decreased mechanical hyperalgesia in DM rats.

The TWL was tested with a thermal stimulator. A significant difference was observed from the 6th week to the 10th week (*p* < 0.01) between the control group and the DM group. At the 10th week, the TWL in the DM+nanoparticle-encapsulated curcumin group was higher than that in the DM group (*p* < 0.01). No difference in the TWL was observed between the DM group and DM+nanoparticle-encapsulated carrier group (*p* > 0.05; Figure 1B). Thus, nanoparticle-encapsulated curcumin decreased thermal hyperalgesia in DM rats.

### Effects of nanoparticle-encapsulated curcumin on the expression of the P2Y12 mRNA and protein in the DRG of the DM rats

**Figure 2:**
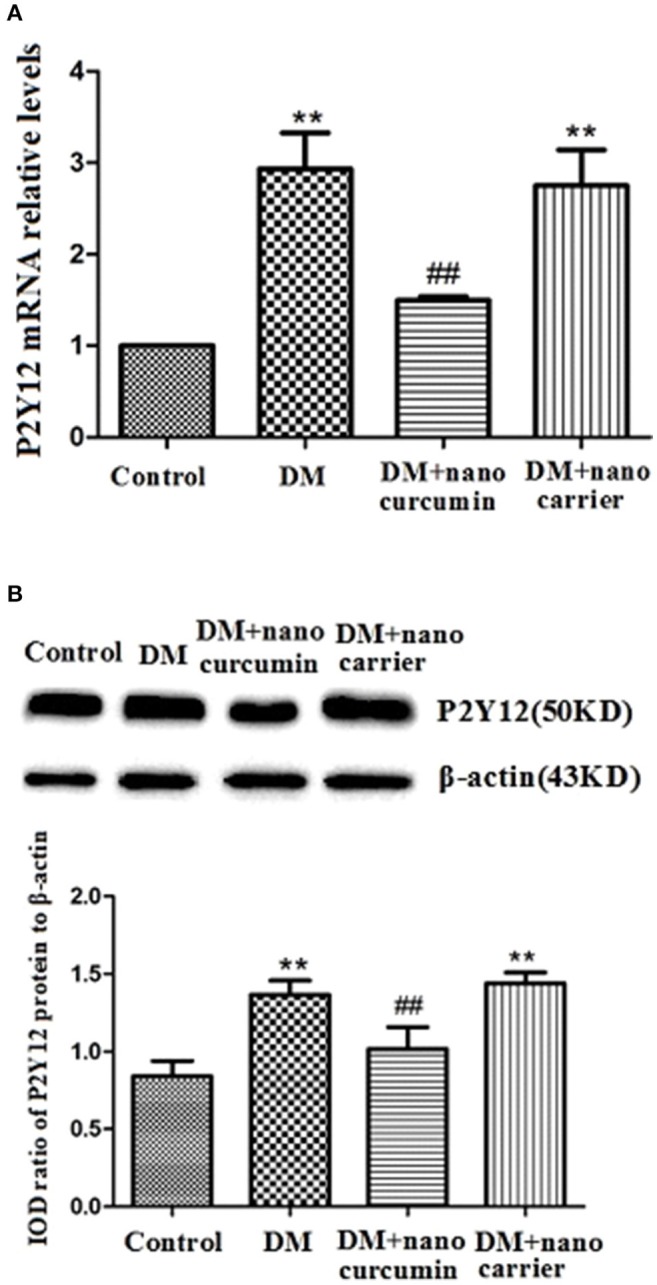
Effects of nanoparticle-encapsulated curcumin on the expression of the P2Y12 mRNA and protein in the DRG of the DM rats. **(A)** The expression of the P2Y12 mRNA in the DRG was measured by RT-qPCR. Expression of the P2Y12 mRNA in the DM group was higher than that in the control group. In the DM+nanoparticle-encapsulated curcumin group, the P2Y12 mRNA expression were significantly decreased compared with those in the DM rats. The experiment was performed three times (*n* = 7 per group). Data are presented as means ± SEMs. ^**^p < 0.01 compared to the control group; ##*p* < 0.01 compared to the DM group. **(B)** The expression of P2Y12 protein in the DRG was assessed by Western blotting. Protein expression was increased in the DM group compared to that in the control group. In the DM+nanoparticle-encapsulated curcumin group, the expression of P2Y12 protein was significantly lower than in the DM group. Bar graphs show the ratio of the levels of the P2Y12 protein to the β-actin protein in each group. Data are displayed as means ± SEMs. ^**^p < 0.01 compared to the ctrl group; ##*p* < 0.01 compared to the DM group.

The expression of the P2Y12 mRNA in the DRG was measured using qPCR. The relative levels of the P2Y12 mRNA in the DM group were significantly increased compared to that in the control group (*p* < 0.01). After the DM group was treated with nanoparticle-encapsulated curcumin, the relative levels of the P2Y12 mRNA were significantly decreased compared to those in the untreated DM group (*p* < 0.01; Figure 2A). No difference in P2Y12 mRNA expression in the DRG was observed between the DM and DM+nanoparticle-encapsulated carrier groups (*p* > 0.05, *n* = 7 per group).

The expression levels of the P2Y12 protein in the DRG were analyzed by Western blotting. According to the image analysis results, levels of the P2Y12 protein (normalized to β-actin levels in the internal control) in the DM group were significantly increased compared to those in the control group (*p* < 0.01). After the DM group was treated with nanoparticle-encapsulated curcumin, the levels of the P2Y12 protein were significantly decreased compared to those in the untreated DM group (*p* < 0.01; Figure 2B). No difference in P2Y12 protein expression in the DRG was observed between the DM and DM+nanoparticle-encapsulated carrier groups (*p* > 0.05, *n* = 7 per group).

Based on these results, the nanoparticle-encapsulated curcumin treatment decreased the expression levels of the P2Y12 mRNA and protein in the DM group.

### Effects of nanoparticle-encapsulated curcumin on the co-expression of P2Y12 and GS in DRG of the DM rats

**Figure 3:**
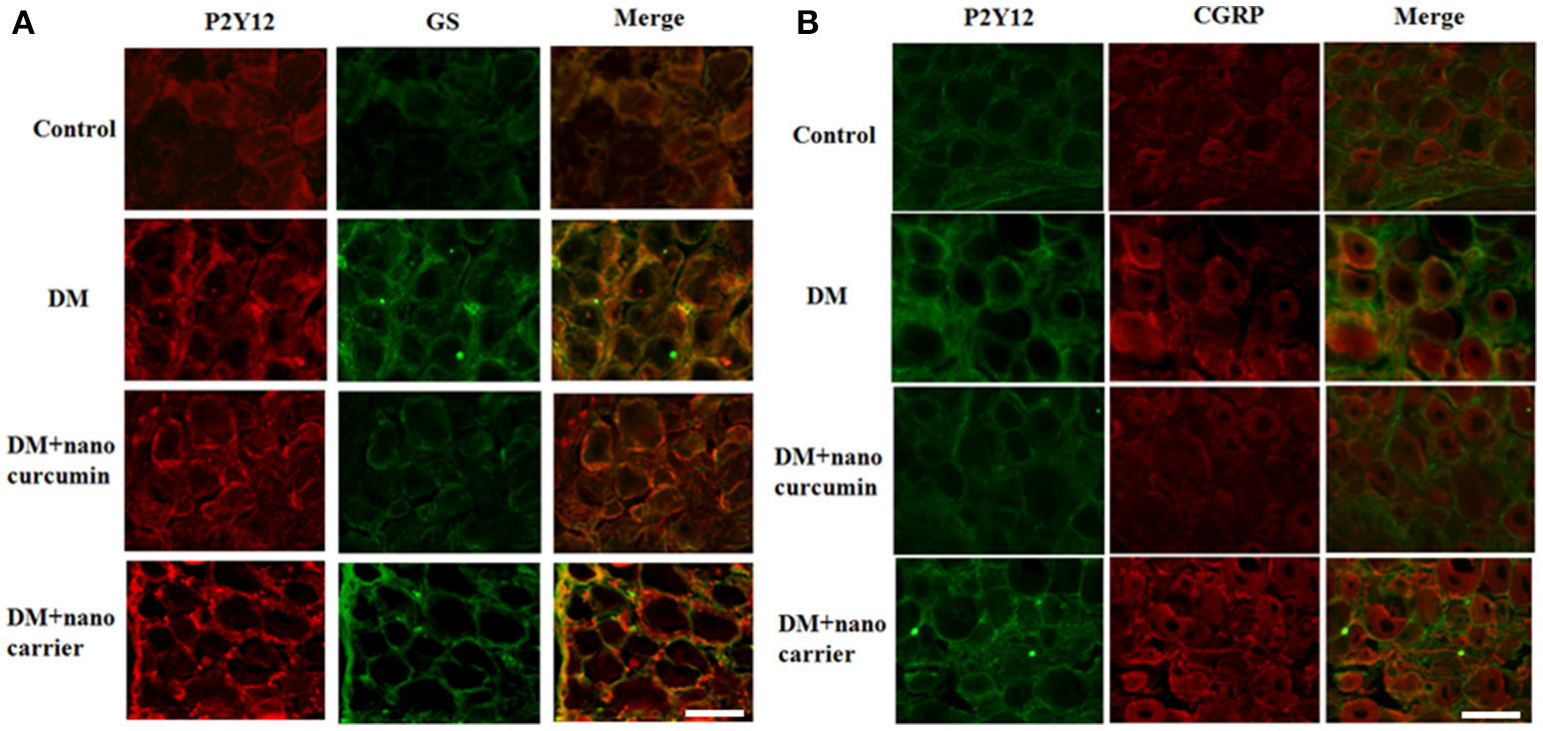
Effects of nanoparticle-encapsulated curcumin on the co-expression of P2Y12 and GS in the DRG of the DM rats. **(A)** Co-expression of P2Y12 and GS in the DRG of DM rats was measured by double-label immunofluorescence staining. Levels of co-expressed P2Y12 and GS in the DM group were higher than those in the control group. The nanoparticle-encapsulated curcumin treatment decreased the co-expression of P2Y12 and GS in DM rats compared with untreated DM rats. No significant differences were observed between the DM group and the DM+nanoparticle-encapsulated carrier group. Scale bar, 30 μm. **(B)** The double-label immunofluorescence of P2Y12 and CGRP was measured by double-label immunofluorescence staining. Co-expression of the P2Y12 receptor and CGRP was observed. The green signal represents P2Y12 staining with FITC, and the red signal indicates CGRP staining with TRITC. The merge represents the P2Y12 and CGRP double staining image. Scale bar, 50 μm.

The co-localization of the P2Y12 receptor and glutamine synthetase (GS) (a marker of SGCs) was measured using double-label immunofluorescence staining. Up-regulated expression of GS is a typical characteristic of SGC activation. The immunofluorescence staining revealed co-localization of the P2Y12 receptor and GS in SGCs in the DRG. More intense staining for the co-localized the P2Y12 receptor and GS was observed in the DM group than that in the control group. The co-expression of the P2Y12 receptor and GS in the DM+nanoparticle-encapsulated curcumin group was significantly decreased compared to that in the DM group. A difference in the co-expression of the P2Y12 receptor and GS was not observed between the DM and DM+nanoparticle-encapsulated carrier groups (Figure 3A). The results revealed the enhanced expression of the P2Y12 receptor on the SGCs in the DRG of the DM rats.

We also detected the expression of P2Y12 and calcitonin gene-related peptide (CGRP, expressed in neurons) by fluorescence double labeling (Figure 3B). The results showed that diabetes up-regulated CGRP expression and nano-curcumin decreased the up-regulation. The results showed that P2Y12 surrounded neuron rather than co-located, which also indicated that P2Y12 receptor was expressed in SGCs rather than neurons.

### Molecular docking of curcumin on the P2Y12 receptor

**Figure 4:**
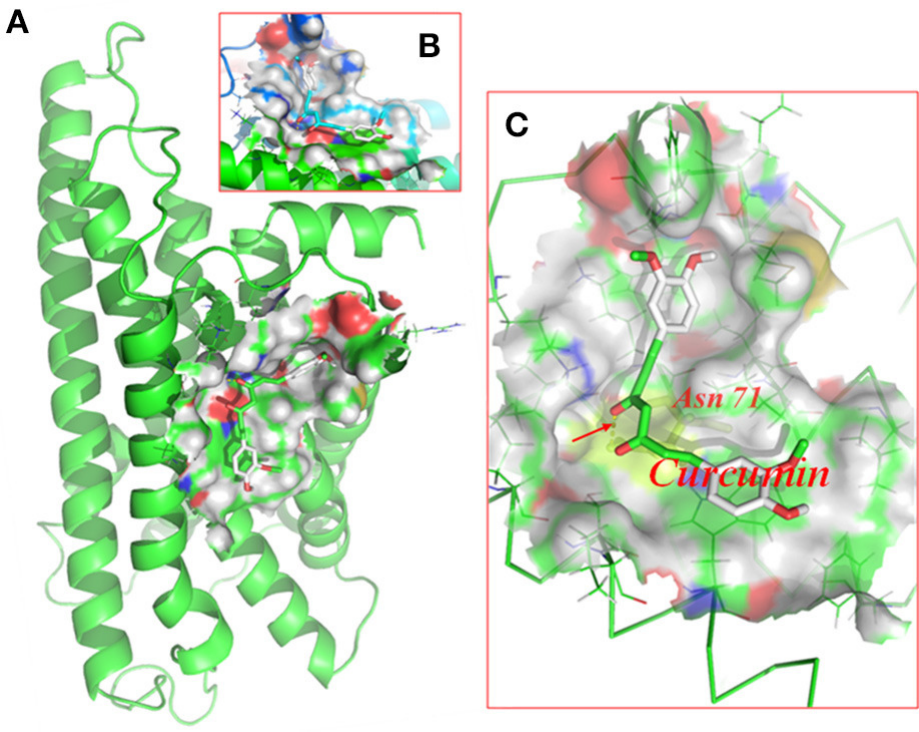
Molecular docking of curcumin on the P2Y12 protein. Simulated model of curcumin docked with the rat P2Y12 protein generated using a computer. Structures in **(A)** (forward map) and **(B)** (top view) show the best docking position for curcumin on the P2Y12 protein. **(C)** The photo indicates the strong binding energy between curcumin and ASN71. Curcumin interacted with rat P2Y12.

Molecular docking of curcumin on a rat P2Y12 protein was performed by AutoDock 4.2. The docking score of the rat P2Y12 protein and curcumin (−7.3, Kcal/mol) showed that curcumin had the perfect fit to interact with the rat P2Y12 receptor (Table 2). The perfect match enabled curcumin to interact with residues in the P2Y12 receptor agonist-binding pocket (Figure 4).

**Table 2:** MOE score of h P2Y12 protein and curcumin (kcal/mol).

**Mode/Rank**	**Affinity (kcal/mol)**	Dist from best mode rmsb^*^ l.b	**Dist from best mode rmse u.b**
1	−7.3	0	0
2	−6.9	29.396	31.651
3	−6.7	29.455	31.991
4	−6.6	28.203	30.642
5	−6.6	5.59	8.838
6	−6.3	34.126	37.371
7	−6.3	28.061	30.567
8	−6.1	29.636	31.47
9	−6.1	29.33	32.803

*The predicted binding affinity is in kcal/mol (Energy)*.

* *rmsd, RMSD values are calculated relative to the best mode and use only movable heavy atoms. Two variants of RMSD metrics are provided, rmsd/lb (RMSD lower bound) and rmsd/ub (RMSD upper bound), differing in how the atoms are matched in the distance calculation*.

### Effects of nanoparticle-encapsulated curcumin on the expression of the interleukin-1β (IL-1β) mRNA and protein in the DRG of the DM rats

**Figure 5:**
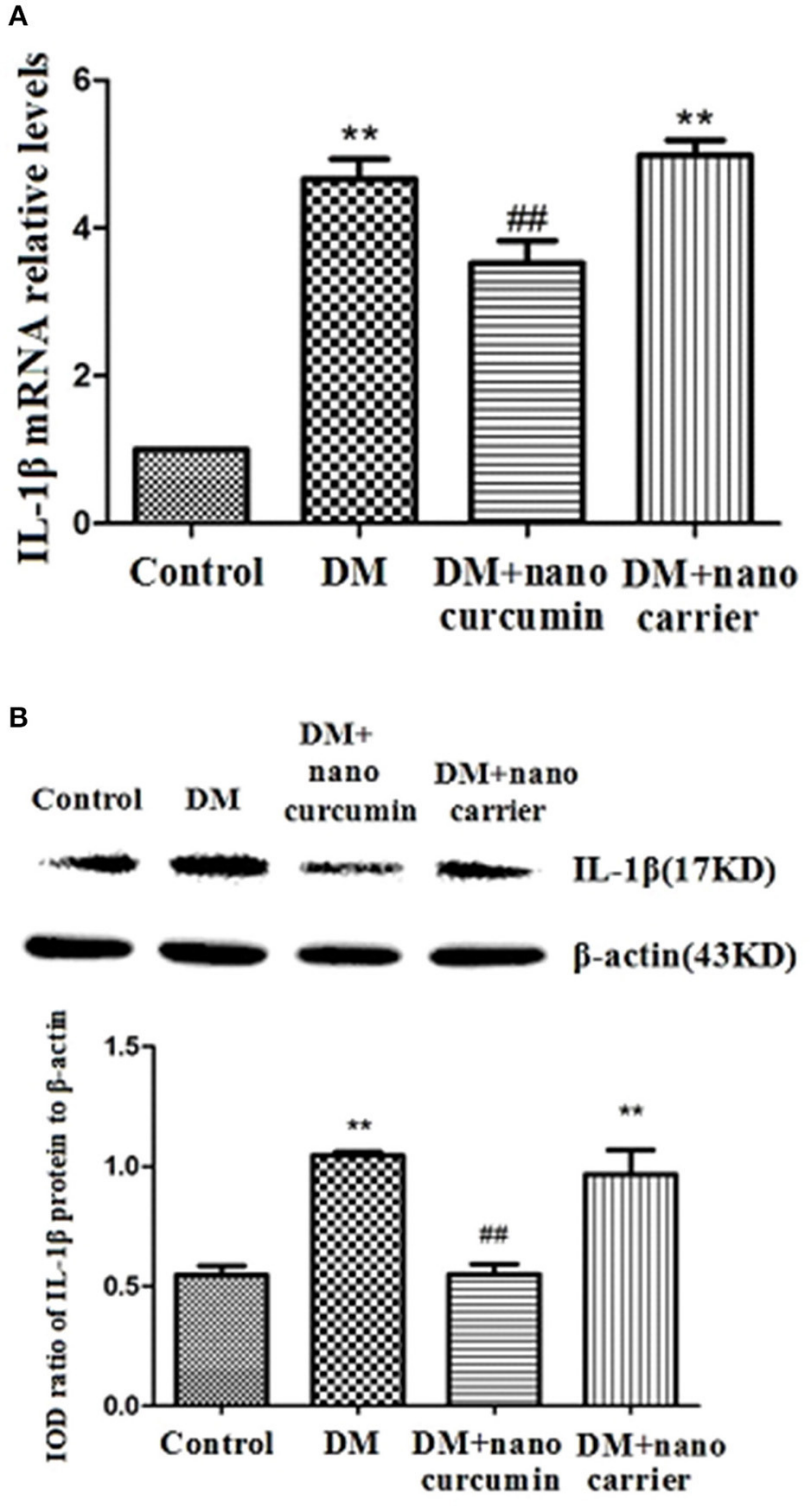
Effects of nanoparticle-encapsulated curcumin on the expression of the interleukin-1β (IL-1β) mRNA and protein in the DRG of the DM rats. **(A)** The expression of the IL-β mRNA in the DRG was measured by RT-qPCR. Expression levels of the IL-β mRNA were higher in the DM group than those in the control group (*p* < 0.01). In DM rats treated with nanoparticle-encapsulated curcumin, the levels of IL-1β mRNA expression were significantly decreased compared with those in the untreated DM rats (*p* < 0.01). The experiment was performed three times (*n* = 7 per group). Data are presented as means ± SEMs. ^**^p < 0.01 compared to the control group; ##*p* < 0.01 compared to the DM group. **(B)** The expression of the IL-β protein in the DRG was measured by Western blotting. Levels of the IL-1β protein were higher in the DM group than those in the control group (*p* < 0.01). In DM rats treated with nanoparticle-encapsulated curcumin, levels of the IL-1β protein were lower than those in the untreated DM group (*p* < 0.01). No difference was observed between the DM+nanoparticle-encapsulated carrier group and the DM group (*p* > 0.05). Bar graphs show the ratio of the IL-1β protein to the β-actin protein in each group. Data are displayed as means ± SEMs (*n* = 7 per group). ^**^p < 0.01 compared to the control group; ##*p* < 0.01 compared to the DM group.

Activation of SGCs in the DRG triggers IL-1β release. The expression of IL-1β mRNA in the DRG was measured by qPCR. Relative levels of the IL-1β mRNA were significantly increased in the DM group compared to those in the control group (*p* < 0.01). Relative levels of the IL-1β mRNA were significantly decreased in the DM+nanoparticle-encapsulated curcumin group compared to those in the DM group (*p* < 0.01; Figure 5A). No difference in IL-1β mRNA expression in the DRG was observed between the DM and DM+nanoparticle-encapsulated carrier groups (*p* > 0.05, *n* = 7 per group).

The expression levels of the IL-1β protein in the DRG were analyzed by Western blotting. According to the image analysis results, levels of the IL-1β protein (normalized to β-actin levels in the internal control) were significantly increased in the DM group compared to those in the control group (*p* < 0.01). The expression levels of the IL-1β protein in the DM+nanoparticle-encapsulated curcumin group were lower than those in the DM group (*p* < 0.01; Figure 5B). No difference in IL-1β protein expression in the DRG was observed between the DM group and the DM+nanoparticle-encapsulated carrier group (*p* > 0.05, *n* = 7 for each group). Thus, the up-regulation of the IL-1β mRNA and protein was related to the active signaling of SGCs in the DRG.

### Effects of nanoparticle-encapsulated curcumin on the expression of the Cx43 mRNA and protein in the DRG of DM rats

**Figure 6:**
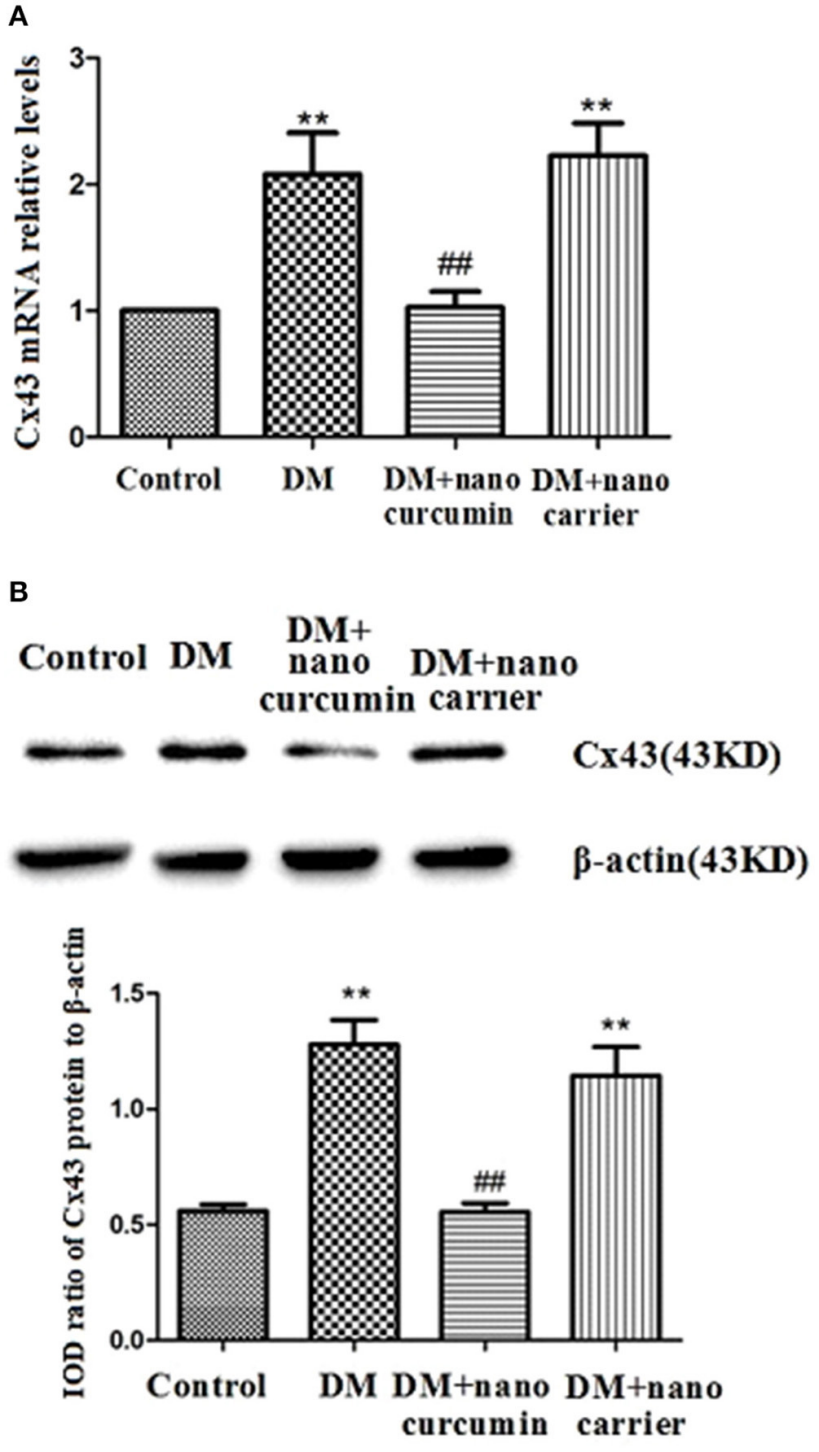
Effects of nanoparticle-encapsulated curcumin on the expression of the Cx43 mRNA and protein in the DRG of the DM rats. **(A)** The expression of the Cx43 mRNA in the DRG was measured by RT-qPCR. Expression levels of the Cx43 mRNA were higher in the DM group than in the control group (*p* < 0.01). In DM rats treated with nanoparticle-encapsulated curcumin, the levels of Cx43 mRNA expression were significantly decreased compared with those in the untreated DM rats (*p* < 0.01). The experiment was performed three times (*n* = 7 per group). Data are presented as means ± SEMs. ^**^p < 0.01 compared to the control group; ##*p* < 0.01 compared to the DM group. **(B)** The expression of the Cx43 protein in the DRG was measured by Western blotting. Expression levels of the Cx43 protein were higher in the DM group than those in the control group (*p* < 0.01). In DM rats treated with nanoparticle-encapsulated curcumin, levels of the Cx43 protein were lower than those in the untreated DM group (*p* < 0.01). No difference was observed between the DM+nanoparticle-encapsulated carrier group and the DM group (*p* > 0.05). Bar graphs show the ratio of the Cx43 protein to the β-actin protein in each group. Data are displayed as means ± SEMs (*n* = 7 per group). ^**^p < 0.01 compared to the control group; ##*p* < 0.01 compared to the DM group.

Connexin 43 (Cx43) is a gap junction subunit and has been shown to be confined to SGCs that to tightly envelop primary sensory neurons (Hanani, 2005; Ohara et al., 2008). An increased correlation between SGCs and up-regulated Cx43 expression has been observed following nerve injury (Hanani, 2005; Ohara et al., 2008). The expression of Cx43 mRNA in the DRG was measured by qPCR. Relative levels of the Cx43 mRNA were significantly increased in the DM group compared to those in the control group (*p* < 0.01). The expression levels of the Cx43 mRNA in the DM+nanoparticle- encapsulated curcumin group were significantly decreased compared to those in the DM group (*p* < 0.01; Figure 6A). No difference in Cx43 mRNA expression in the DRG was observed between the DM and DM+nanoparticle-encapsulated carrier groups (*p* > 0.05, *n* = 7 per group).

The expression levels of the Cx43 protein in the DRG were analyzed by Western blotting. According to the image analysis results, levels of the Cx43 protein (normalized to β-actin levels in the internal control) were significantly increased in the DM group compared to those in the control group (*p* < 0.01). Levels of the Cx43 protein in the DM+nanoparticle-encapsulated curcumin group were lower than in the DM group (*p* < 0.01; Figure 6B). No difference in Cx43 protein expression in the DRG was observed between the DM and DM+nanoparticle-encapsulated carrier groups (*p* > 0.05, *n* = 7 per group). Thus, the nanoparticle-encapsulated curcumin treatment decreased the up-regulated expression of the Cx43 mRNA and protein in the DRG of the DM rats and might reduce the increased interactions between SGCs.

### Effects of nanoparticle-encapsulated curcumin on p-AKT levels in the DRG of DM rats

**Figure 7:**
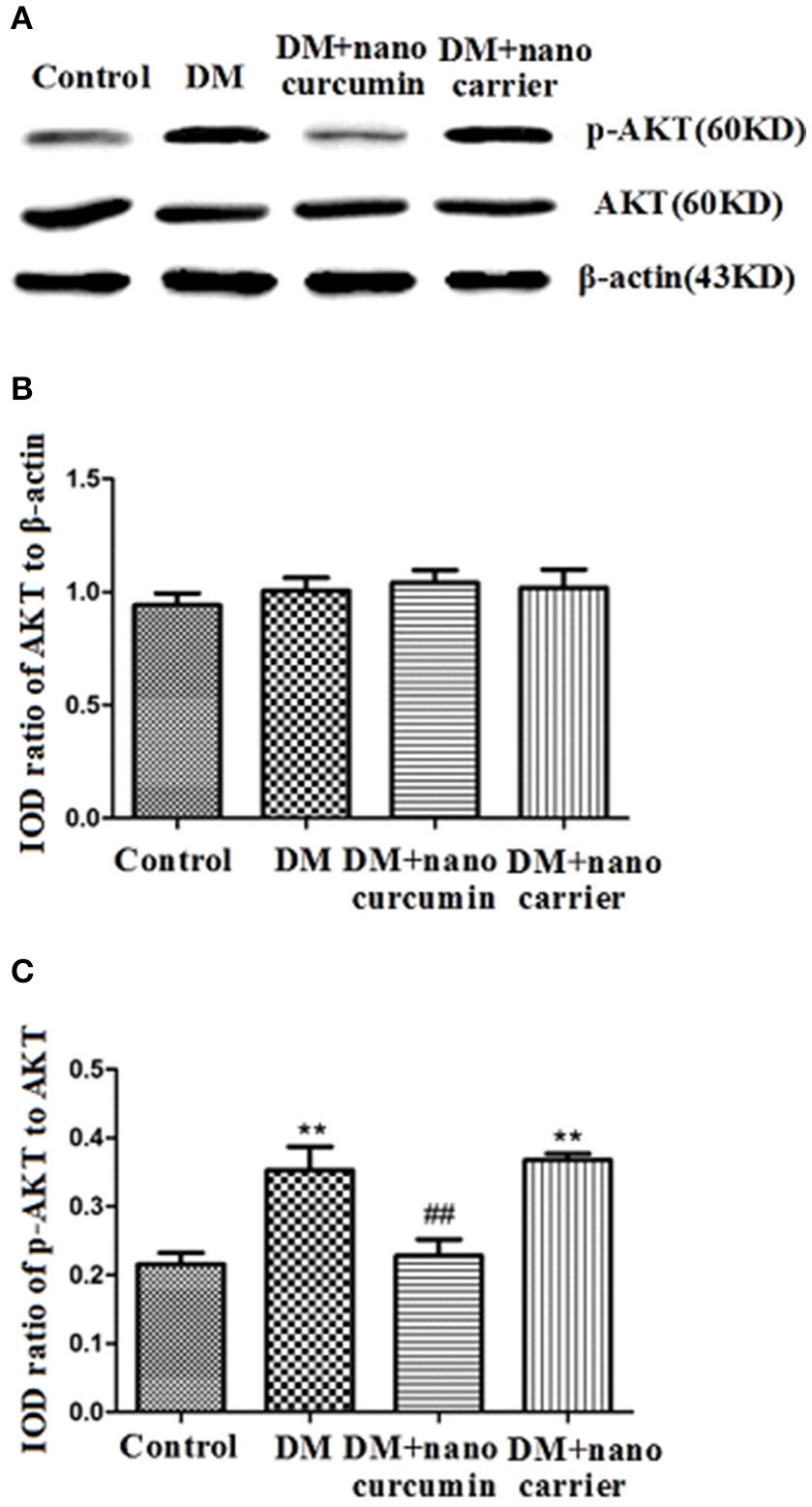
Effects of nanoparticle-encapsulated curcumin on p-AKT levels in the DRG of DM rats. **(A,B)** Ratios of Akt to β-actin integrated optical densities (IODs) were not significantly different between the DM group and the control groups (*p* > 0.05). **(A,C)** The ratio of the p-Akt to Akt IODs was higher in the DM group than that in the control group (*p* < 0.01, *n* = 7 for each group). The ratio of the p-AKT to AKT IODs was significantly increased in the DM group compared to that in the control group (*p* < 0.01). **(A,C)** The ratio of p-Akt to Akt IODs in DM rats treated with nanoparticle-encapsulated curcumin was significantly decreased compared with that in the untreated DM group (*p* < 0.01, *n* = 7 per group). Data are displayed as means ± SEMs, *n* = 7. ^**^p < 0.01 compared to the ctrl group; ##*p* < 0.01 compared to the DM group.

Akt phosphorylation is a marker of activated Akt. Akt phosphorylation is related to neuropathic pain mechanisms (Sun et al., 2006; Xu et al., 2007; Guedes et al., 2008). Levels of the AKT and p-AKT proteins in the DRG were analyzed by Western blotting. According to the image analysis results, significant differences in the ratios of the AKT to β-actin IODs were not observed between the DM group and control group (*p* > 0.05; Figures 7A,B). The ratio of the p-AKT to AKT IODs was significantly increased in the DM group compared to that in the control group (*p* < 0.01;Figures 7A,C). After the DM rats were treated with nanoparticle-encapsulated curcumin, the ratio of the p-AKT to AKT IODs was significantly decreased compared with that in the untreated DM rats (*p* < 0.01; Figures 7A,C). A difference in the ratio of the p-AKT to AKT IODs in the DRG was not detected between the DM and DM+nanoparticle-encapsulated carrier groups (*p* > 0.05, *n* = 7 per group). Based on these results, the nanoparticle-encapsulated curcumin treatment inhibited AKT activation and decreased hyperalgesia in DM rats.

## Discussion

Dorsal root ganglia (DRG) neurons are the pseudo-unipolar afferent neurons responsible for transmitting primary sensory information from the periphery to the central nervous system. The DRG plays an important role in the processing and transmission of DNP signals (Hanani et al., 2014; Liu et al., 2016; Li et al., 2017; Peng et al., 2017). Based on our results, mechanical and thermal hyperalgesia were increased in DM rats. Meanwhile, the expression of P2Y12 receptor was increased in the DRG of DM rats compared with that in control rats. The P2Y12 receptor is related to the pathological changes in inflammatory and lingual neuropathic pain, diabetes, chronic kidney disease, cardiovascular diseases and stroke (Burnstock and Novak, 2012; Katagiri et al., 2012; Burnstock, 2013; Magni and Ceruti, 2013; Horvath et al., 2014; Engwenyu et al., 2017). Nanoparticle-encapsulated curcumin treatment decreased the blood glucose levels in type 2 diabetic rats. In addition, DM rats treated with nanoparticle-encapsulated curcumin exhibited decreased expression levels of the P2Y12 mRNA and protein accompanied by increased MWTs and TWLs. The nanoparticle-encapsulated curcumin treatment decreased the transmission of DNP signals by the P2Y12 receptor in the DRG of DM rats.

After nerve injury, the activation of SGCs enwrapping the neuronal soma results in neuropathic pain (Hanani, 2005; Takeda et al., 2009; Hanani et al., 2014; Costa and Moreira Neto, 2015; Liu et al., 2016). Elevated expression of GS (a marker of SGCs) is a marker of activated SGCs in the DRG. In addition, the levels of co-expressed P2Y12 and GS were increased in the DM group compared with the control group. The nanoparticle-encapsulated curcumin treatment decreased the co-expression of P2Y12 and GS in DM rats. The results for the co-expression of P2Y12 and CGRP (expressed in neurons) tested by fluorescence double labeling (Figure 3B) showed that diabetes up-regulated CGRP expression and nano-curcumin decreased the up-regulation. P2Y12 surrounded neuron rather than co-located, which also indicated that P2Y12 receptor was expressed in SGCs rather than neurons. Therefore, the nanoparticle-encapsulated curcumin treatment decreased the expression levels of the P2Y12 receptor in SGCs in the DRG and reduced mechanical and thermal hyperalgesia in DM rats. The photo in Figure 4 shows that curcumin interacted with rat P2Y12. Interaction energies for the docked complexes were calculated by AutoDock 4 and are shown in Table 1. In Table 1, a higher value for the negative interaction energy indicates a more efficient interaction between the rat P2Y12 receptor and curcumin. Curcumin may bind to the P2Y12 protein to limit the interaction between the P2Y12 receptor and its agonist, leading to the inhibition of the P2Y12 receptor. Based on our results, curcumin might exert an inhibitory effect on the P2Y12 receptor.

In response to a peripheral injury, activation of SGCs increases the production of cytokines such as IL-1β (Hanani, 2005, 2012; Costa and Moreira Neto, 2015). Up-regulation of the P2Y12 receptor in DM rats was followed by increased expression of the IL-1β mRNA and protein. SGC modulates the excitability of primary sensory neurons via IL-1β (Hanani, 2005, 2012; Costa and Moreira Neto, 2015). The up-regulation of the IL-1β protein in the DRG increased the mechanical and thermal hyperalgesia in DM rats compared to that in control rats. P2Y12 receptor activation increases IL-1β release in neuropathic pain models (Hanani, 2005, 2012; Horvath et al., 2014). Curcumin has anti-inflammatory effects and has been reported to partially ameliorate DNP and the effects of nanoparticles curcumin in 4 mg/kg doses is equate to that in the 50–200 mg/kg doses of conventional curcumin (Sharma et al., 2007; Li et al., 2013; Banafshe et al., 2014; Zhao et al., 2014). The nanoparticle-encapsulated curcumin treatment decreased the levels of P2Y12 and IL-1β in the DRG and reduced the mechanical and thermal hyperalgesia in DM rats. Thus, nanoparticle-encapsulated curcumin treatment reduced IL-1β expression and decreased neuropathic pain behaviors mediated by the P2Y12 receptor.

Gap junctions between SGCs have an important role in neuronal excitability (Hanani, 2005, 2012; Costa and Moreira Neto, 2015). After nerve injury, the density (number) of gap junctions and coupling between sensory ganglia SGCs increase in models of chronic pain (Hanani, 2005, 2012; Ohara et al., 2008; Ledda et al., 2009; Huang et al., 2010). In the present study, the expression of the Cx43 mRNA and protein was increased in the DM group compared with the control group. The up-regulated expression of Cx43 contributes to the induction and/or maintenance of pain (Hanani, 2005, 2012; Ohara et al., 2008; Ledda et al., 2009). The nanoparticle-encapsulated curcumin treatment decreased the up-regulation of Cx43 mRNA and protein in DM rats. The increased ATP levels observed after injury are related to the increased numbers of gap junctions between SGCs and influence the excitability of neighboring neurons (Hanani, 2005, 2012; Costa and Moreira Neto, 2015). Nerve damage or inflammation increases the sensitivity of P2Y receptors to ATP mediated (Hanani, 2005, 2012; Costa and Moreira Neto, 2015). Thus, the nanoparticle-encapsulated curcumin treatment decreased the DM-induced increase in the Cx43 levels in the DRG and inhibited ATP signaling mediated by the P2Y12 receptor in DM rats. The up-regulation of P2Y12 on SGCs and the up-regulation of the IL-1β and Cx43 in the DRG indicated the activation of SGCs in the DRG. The nano-curcumin treatment inhibited the activation of SGCs accompanied by its anti-inflammatory effect to decrease the up-regulated CGRP expression in the DRG neurons.

Blockade of protein kinase B/Akt activation decreases pain behaviors in neuropathic pain models (Sun et al., 2006; Xu et al., 2007; Guedes et al., 2008). The up-regulation of the P2Y12 receptor is related to Akt activation (Irino et al., 2008). Based on our data, the ratio of p-Akt to Akt IODs in DM rats was higher than that in control rats. After DM rats were treated with nanoparticle-encapsulated curcumin, the ratios of p-Akt to Akt IODs were significantly reduced compared with those in untreated DM rats. The nanoparticle-encapsulated curcumin treatment decreased the up-regulation of the P2Y12 receptor and p-Akt levels in the DRG of DM rats. Notably, p-Akt is a marker of Akt activation. The dose (50–200 mg/kg) in conventional way of administration of curcumin is very higher than that in nanoparticle-encapsulated curcumin treatment (Sharma et al., 2007; Li et al., 2013; Banafshe et al., 2014; Zhao et al., 2014). Nanoparticle-encapsulated curcumin was used to enhance bioavailability. Therefore, the nanoparticle-encapsulated curcumin treatment reduced P2Y12 receptor-mediated Akt activation in the DRG, thereby relieving mechanical and thermal hyperalgesia in DM rats.

In conclusion, diabetic peripheral neuropathy increased the expression levels of the P2Y12 receptor on SGCs in the DRG and enhanced mechanical and thermal hyperalgesia in DM rats. Up-regulation of the P2Y12 receptor on SGCs in the DRG increased the production of pro-inflammatory cytokines. Up-regulation of IL-1β and Cx43 expression increased neuronal excitability in the DRG and resulted in mechanical and thermal hyperalgesia in DM rats. The nanoparticle-encapsulated curcumin treatment decreased the up-regulated P2Y12 expression levels in the DRG, reduced the up-regulation of IL-1β and Cx43, and decreased p-Akt levels in the DRG of DM rats. Therefore, the nanoparticle-encapsulated curcumin treatment decreased the up-regulation of the P2Y12 receptor on SGCs in the DRG and decreased mechanical and thermal hyperalgesia in DM rats.

## Author contributions

Conceived and designed the experiments, contributed reagents, materials, and analysis tools: SLiang and GL. Performed the experiments: TJ, JR, GL, LZ, SZ, ZY, BW, LL, HY, LS, CZ, YG, SLiu, HX, and HL. Analyzed the data: TJ and GL. Wrote the article: TJ, GL, and SLiang. The article was revised by SLiang and GL. The study idea was from SLiang.

### Conflict of interest statement

The authors declare that the research was conducted in the absence of any commercial or financial relationships that could be construed as a potential conflict of interest.
